# Supplementary material for: Doping-induced perturbation and percolation in the two-dimensional Anderson lattice
Source: Sci Rep. 2017 Apr 6;7:46089. doi: 10.1038/srep46089 (PMC5382687; doi:10.1038/srep46089)
Supplement: Supplementary Information [file srep46089-s1.pdf]

Supplementary Information: Doping-induced perturbation and percolation in the two-dimensional Anderson lattice

Lan-ying Wei[1] **and Yi-feng Yang**[1,2,3]

[1]Beijing National Laboratory for Condensed Matter Physics and Institute of Physics, Chinese Academy of Sciences, Beijing 100190, China
[2]Collaborative Innovation Center of Quantum Matter, Beijing 100190, China
[3]School of Physical Sciences, University of Chinese Academy of Sciences, Beijing 100190, China

Here we provide further analysis on the finite size effect and the critical scaling behavior of the *dc* conductivity.

Figure S1 compares the difference in the *dc* conductivity, $\sigma_{dc}(\beta t = 8) - \sigma_{dc}(\beta t = 4)$, for $L = 6, 8, 10, 12$. We see that the critical doping is almost unchanged with increasing lattice size L. We note that the extrapolation at $x = 0.99$ cannot be trusted as the doping is actually $(L^2 - 1)/L^2$, which varies with L. Similar variations exist for other doping, but are less crucial.

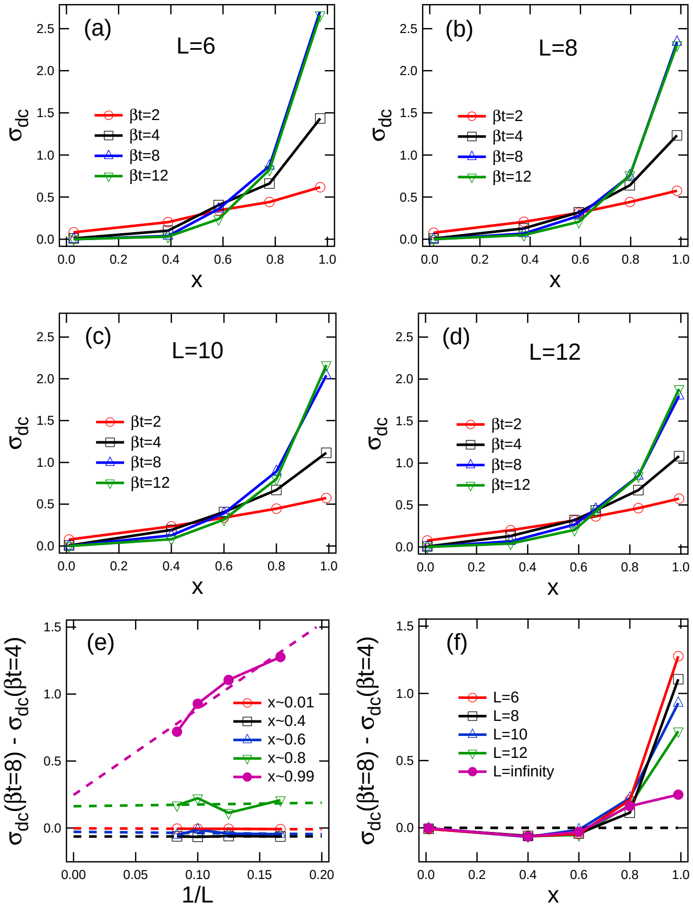

Figure S1. Lattice size dependence of the *dc* conductivity σ_{dc}. (a-d) σ_{dc} as a function of doping x at different temperatures for the lattice size $L = 6$, 8, 10, and 12. (e) $\sigma_{dc}(\beta t = 8) - \sigma_{dc}(\beta t = 4)$ as a function of L for different dopings, showing the size dependence of the metallic or insulating behavior. The dashed lines illustrate linear extropolations to the numerical data. (f) Doping dependence of $\sigma_{dc}(\beta t = 8) - \sigma_{dc}(\beta t = 4)$ for different lattice sizes, showing slight change in the critical doping $x_c \approx 0.6$ for the insulator-to-metal transition.

Figure S2 shows a power law fit to the dc conductivity, $\sigma_{dc} - \sigma_{dc}(x_c)$, with $(x - x_c)^{\mu}$ for $L = 12$ and $\beta t = 12$, 14 and 16. The fit yields $\mu = 1.67$, 1.56 and 1.44, respectively, while previous theories have predicted $\mu \approx 1.3$ for zero temperature and 2D infinite lattice. Our results are close to the predicted values.

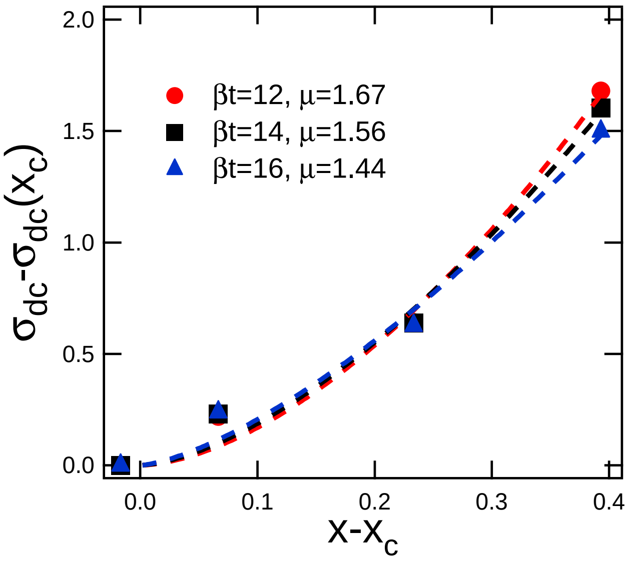

Figure S2. Fit to the dc conductivity, $\sigma_{dc} - \sigma_{dc}(x_c)$, with $(x - x_c)^{\mu}$ for $L = 12$ and $\beta t = 12$, 14 and 16.
